# RUST-ED BEYOND REPAIR – LET’S TALK INTERNET GAMING AND ADDICTION

**DOI:** 10.1192/j.eurpsy.2023.1382

**Published:** 2023-07-19

**Authors:** K. Laxton

**Affiliations:** University of Witwatersrand, University, Johannesburg, South Africa

## Abstract

**Introduction:**

“Rust is one of the cruellest games on Steam, and that’s what makes it so compelling.” (PC Gamer) Rust is a multiplayer-only survival video game. It’s full release was in 2018. The average age of a player is between 17 and 40 years. However, one must question the age group that enters the game and continues to play. This presentation will focus on adolescence, gaming addiction, insomnia, and the developing brain.

**Objectives:**

Rust is a game of round-the-clock survival in the wilderness whereby players must manage the basic essence of human need to “stay alive”. However, it requires a player to be present for a consistent duration of time, or risk “dying”. What does this mean for an adolescent, entering their final two years of school? What is their focus? How does this potentially affect the development of the young mind, still vulnerable to an immediate (real) world moulding a brain from adolescence to young adulthood?

**Methods:**

Questions regarding adolescent epigenetics, insomnia and addiction erupt in mental health when Rust and other international multiplayer survival games capture and imprison the mind of our youth.

**Results:**

The WHO added “gaming disorder” to the 2018 *International Classification of Diseases.* But the APA manual, the DSM-5, did not. The University of New Mexico suggests that 6-15% of all gamers exhibit signs and symptoms consistent of addiction. In an article published in February 2019 BioPsychoSocial Medicine states: ‘Across studies, the presence of International Gaming Disorder (IGCD had a negative effect on sleep and schoolwork in minors … Brain imaging studies indicate that impaired cognitive control in minors with IGD is associated with abnormal function in the prefrontal cortex and striatum.’

**Conclusions:**

Today we ask: of the 83.5% of online gamers and 3.9% of youth reporting problematic behaviour are we Rusted beyond Repair?

**Disclosure of Interest:**

None Declared

